# Association of anti-oxidative capacity of HDL with subclinical atherosclerosis in subjects with and without non-alcoholic fatty liver disease

**DOI:** 10.1186/s13098-021-00741-5

**Published:** 2021-10-26

**Authors:** Sara Karami, Hossein Poustchi, Negar Sarmadi, Amir Reza Radmard, Fatemeh Ali Yari, Abbas Pakdel, Parisa Shabani

**Affiliations:** 1 Department of Biochemistry, School of Medicine, Semnan University of Medical Sciences, Semnan, Iran; 2 Liver and Pancreatobiliary Diseases Research Center, Digestive Diseases Research Institute, Tehran University of Medical Sciences, Tehran, Iran; 3 Department of Radiology, Shariati Hospital, Tehran University of Medical Sciences, Tehran, Iran; 4 Nervous System Stem Cells Research Center, Semnan University of Medical Sciences, Semnan, Iran; 5 Department of Integrative Medical Sciences, Northeast Ohio Medical University, Rootstown, OH USA

**Keywords:** HDL oxidative index, Non-alcoholic fatty liver disease, Cardiovascular disease, Carotid intima-media thickness

## Abstract

**Background:**

Non-alcoholic fatty liver disease (NAFLD) patients are at a substantial risk for developing cardiovascular disease (CVD). High-density lipoprotein (HDL) is well known to have protective effects against the development of atherosclerotic CVD. One of the major antiatherogenic effects of HDL is its anti-oxidative function.

**Objectives:**

This study investigated the association of anti-oxidative capacity of HDL with subclinical atherosclerosis in NAFLD and non-NAFLD subjects.

**Methods:**

A total of 143 subjects including 51 NAFLD and 92 control subjects were included in this case–control study. HDL oxidative index (HOI) was determined spectrophotometrically using a cell-free method in the presence of a fluorescent substrate dichlorofluorescein diacetate (DCFDA). Paraoxonase 1 (PON1) activity, superoxide dismutase (SOD) activity, and malondialdehyde (MDA) plasma levels were assessed in both groups.

**Results:**

The NAFLD patients with impaired HDL anti-oxidative function (HOI  ≥ 1) had higher MDA levels, aspartate amino transferase (AST), liver stiffness (LS), and carotid intima-media thickness (cIMT) values compared to the controls. HDL oxidative index (HOI) was positively correlated with MDA levels and cIMT and negatively correlated with SOD activity.

**Conclusions:**

Higher circulating levels of MDA were associated with the impaired anti-oxidative function of HDL in NAFLD. The impaired anti-oxidative capacity of HDL might be related to NAFLD severity and subclinical atherosclerosis in NAFLD patients.

**Supplementary Information:**

The online version contains supplementary material available at 10.1186/s13098-021-00741-5.

## Introduction

Non-Alcoholic Fatty Liver Disease (NAFLD) is the most leading cause of chronic liver disease which affects an estimated 20–30% of the general population [1]. NAFLD is strongly associated with other metabolic conditions such as obesity, type 2 diabetes mellitus, and cardiovascular diseases [1]. Despite liver-related morbidity and mortality, cardiovascular disease (CVD) is the most important cause of mortality in the NAFLD population [2].

NAFLD patients exhibit atherogenic dyslipidemia with low levels of high-density lipoprotein cholesterol (HDL-C) [3]. Several studies demonstrated an association of higher plasma high-density lipoprotein (HDL) cholesterol levels with a lower risk of atherosclerotic cardiovascular disease (CVD) morbidity and mortality. However, recent studies suggest that HDL particle functionality might be a better predictor of CDV risk than HDL cholesterol mass levels [4]. HDL particle has numerous antiatherogenic functions. One of the important anti-atherogenic properties of HDL is its anti-oxidative function, the ability to suppress LDL oxidation which in turn decreases the atherogenicity of LDL particles [5].

NAFLD presence was shown to be associated with the size and functional heterogeneity of HDL particles [6, 7]. NAFLD patients had an abnormal distribution of HDL subpopulations and the lipid composition of HDL particles [7]. Proteomics analysis showed altered protein composition of HDL particles in the NAFLD patients [8]. HDL size differed by fatty liver index and NAFLD patients had lower levels of larger HDL particles [6]. Association of HDL ApoA1 with liver fat content was only found in the HDL lacked apoC3 [9]. This heterogeneity results in the alteration of different HDL functionalities such as anti-oxidative function. Several studies addressed the anti-oxidative function of HDL in CVD and other disorders with high cardiovascular risk [10–13]. Moreover, previous studies showed impaired HDL cholesterol efflux capacity in NAFLD [14, 15]. However, no study evaluated the anti-oxidative function of HDL in NAFLD patients.

Oxidative stress which occurs because of the imbalance between antioxidants and reactive oxygen species (ROS) is involved in the initiation and promotion of NAFLD [16, 17]. One of the mechanisms which cause HDL dysfunction is a modification with lipid peroxidation products such as malondialdehyde (MDA) [18, 19]. It has been shown that MDA impaired HDL’s athero-protective functions. MDA generates lysine adducts on apoA-1 and blocks the cholesterol efflux function of HDL [20]. Incubation of macrophages with MDA modified HDL also led to an increased ability to generate ROS [21]. The antioxidant enzymes including superoxide dismutase and PON1 diminish ROS and lipid peroxidation. Paraoxonase 1 (PON1) associates with HDL and catalyzes the hydrolysis of lipid peroxides [22].

Increasing data have demonstrated that NAFLD patients have abnormal circulating markers of oxidative stress, such as increased malondialdehyde (MDA), superoxide dismutase activity, and PON1 activity [23, 24]. Having a disturbed anti-oxidative system along with a high risk of CVD in these patients suggests that impaired antioxidant activities of HDL, as an important player in CVD, may be the potential link between these phenomena. In this study, we aimed to investigate differences in HDL antioxidative capacity between NAFLD patients and controls. Moreover, we sought to compare carotid intima-media thickness as well as other relevant biomarkers between NAFLD patients and controls with preserved and reduced HDL antioxidative capacity.

## Material and methods

### Study participants

In the current study, 92 Controls and 51 NAFLD patients were selected from Golestan Cohort Study [25]. The study was a case–control study and approved by the medical ethics committee of the Semnan University of Medical Sciences (Ethics Code: IR.SEMUMS.REC.1397. 331) and all participants gave written informed consent. All participants were male, aged 50–81 years old. The diagnosis of NAFLD was based on abdominal ultrasonography. Subjects were excluded if they met one of the following criteria: a history of alcohol consumption (> 30 g/d), diabetes, viral hepatitis, autoimmune liver disease, hemochromatosis, Wilson’s disease. None of the patients were taking medication that has been reported to induce hepatitis, statins and antioxidants.

### Ultrasonography and elastography

Ultrasound assessment was performed using an Accuvix XQ ultrasound unit (Medison, South Korea) equipped with a 3–7 MHz curved array and a 5–12 MHz linear array transducer for the evaluation of liver, abdominal fat, and carotid arteries as previously described [25]. Visceral Adipose Tissue thickness (VAT), carotid intima-media thickness (cIMT), and liver stiffness (LS) were measured as described before [25].

### Anthropometric and laboratory evaluations

Anthropometric parameters were measured under standardized protocols. Fasting blood samples were obtained from the participants following overnight fasting. Serum and plasma were isolated and frozen in aliquots at  − 80 °C for further analysis. Biochemical parameters including Fasting blood glucose (FBG), serum total cholesterol (TC), triglycerides (TG), high-density lipoprotein cholesterol (HDL-C), low-density lipoprotein cholesterol (LDL-C), and levels of alanine amino transferase (ALT), aspartate amino transferase (AST), gamma glutamyl transferase (GGT) were measured by automated enzymatic methods using commercial kits (Pars Azmoon, Iran).

Paraoxonase 1 (PON1) enzymatic activity was measured according to a method described before. Paraoxon (Sigma-Aldrich, Germany) was used as the substrate and PON1 activity was assessed by measuring the rate of substrate hydrolysis to p-nitrophenol. The formation of p-nitrophenol was recorded at 412 nm and activity was expressed as μmol p-nitrophenol/L/plasma/min [26]. Superoxide dismutase (SOD) activity was measured by a SOD activity assay kit (Teb Pazhouhan Razi, Iran) following the manufacturer’s instructions. Plasma MDA levels were determined using an MDA assay kit (Teb Pazhouhan Razi, Iran) following the manufacturer’s instructions.

### Measurement of high‐density oxidative index (HOI)

HOI measures the ability of apoB-depleted plasma to inhibit LDL oxidation in the presence of dichlorofluorescein diacetate (DCFDA) (Sigma-Aldrich, Germany). A cell-free assay was performed as previously described with some modifications [5]. Before analysis, apolipoprotein (apo) B depleted plasma was prepared as previously described [5]. LDL was diluted in PBS to a final cholesterol concentration of 100 μg/mL and oxidized by CuSO4 (100 μmol/L) for 6 h at 37 °C. Oxidized LDL with a concentration of 1.4 μg/ml, DCFDA with a concentration of 0.725 μg/ml and apo B-depleted plasma from the participants with PBS to a final volume of 175 μl were added and incubated at 37 °C for 60 min. Fluorescence intensity was measured with an excitation wavelength of 485 nm and emission wavelength of 530 nm using the synergy h1 hybrid multi-mode microplate reader (BioTek, USA). The HDL oxidative index (HOI) was calculated as the ratio of fluorescence intensity in the presence of apo‐B‐depleted plasma samples divided by the fluorescence intensity in the absence of apo‐B‐depleted plasma. An HOI  < 1 was considered as anti-oxidative HDL function and an HOI  ≥ 1 was considered as pro‐oxidative HDL.

### Statistical analysis

The Kolmogorov‐Smirnov test was used to test for the normal distribution of data. Categorical data are presented as percentages, continuous data as the mean  ±  SD or median (IQR) as appropriate. Comparison between two groups was performed using the independent-sample t-test or Mann‐Whitney U test for continuous data and the chi‐squared test for categorical data. Differences among the four subgroups as classified based on the presence of NAFLD and HOI were determined using ANOVA followed by Tukey’s post hoc tests. Pearson correlation was applied to determine the correlations between HOI and the other parameters in the whole study population. Non-normally distributed data were log-transformed before ANOVA and Pearson correlation tests. Significant differences were defined by P  <  0.05 (*) or (#), P  <  0.01 (**) or (##), and P  <  0.0001 (****) or (####). All of the statistical analyses were performed using the IBM SPSS Statistic 27 and GraphPad prism 9.

## Results

Basic clinical and laboratory characteristics of the study groups are presented in Table 1. BMI, waist circumference (WC), waist-to-hip ratio, fasting blood glucose (FBG), TG, visceral fat, systolic blood pressure, diastolic blood pressure, AST, ALT, GGT, and LS were significantly higher in the NAFLD group compared with the control group. NAFLD patients had lower HDL-C than the control subjects. There was not any significant difference in age, total cholesterol (TC), LDL-C, and cIMT between the two groups.

**Table 1:** Baseline characteristics comparison between NAFLD patients and control subjects

Parameter	Control (n = 92)	NAFLD (n = 51)	P value
Age (years)	59.0 (55.0–66.0)	56.0 (54.0–61.0)	0.273
BMI (kg/m^2^)	24.8 ± 3.6	29.3 ± 3.4	< 0.001
WC (cm)	93.2 ± 11.1	103.6 ± 9.6	< 0.001
WHR	0.97 (0.92–1.00)	0.98 (0.94–1.03)	0.002
Weight (kg)	68.7 (61.0–79.5)	80.7 (73.5–89.0)	< 0.001
TC (mg/dL)	205.5 ± 39.4	207.3 ± 37.0	0.779
LDL-C (mg/dL)	121.6 ± 33.7	124.6 ± 28.7	0.594
HDL-C (mg/dL)	57.4 ± 12.5	51.9 ± 11.1	0.007
TG (mg/dL)	112.0 (82.0–158.0)	148.5 (112.5–186.0)	0.016
Systolic blood pressure (mmHg)	131.5 ± 21.3	144.9 ± 23.7	0.001
Diastolic blood pressure (mmHg)	79.9 ± 11.0	86.8 ± 12.4	0.001
FBG (mg/dL)	92.71 ± 9.00	99.57 ± 11.21	< 0.001
Visceral fat (%)	46.5 ± 20.7	70.2 ± 18.0	< 0.001
AST (U/L)	18.5 (15.0–22.0)	22.0 (19.5–27.0)	< 0.001
ALT (U/L)	15.5 (11.0–22.0)	31.5 (23.0–41.0)	< 0.001
GGT (U/L)	22.37 (17.40–29.10)	28.65 (23.53–38.50)	< 0.001
LS (kPa)	3.8 (3.3–4.3)	5.0 (4.2–6.6)	< 0.001
cIMT (mm)	0.8 (0.75–0.87)	0.81 (0.74–0.91)	0.863
HOI	0.92 ± 0.19	0.95 ± 0.24	0.405
HOI ≥ 1 frequency (%)	33.69	41.17	0.468
SOD (U/mg protein)	10.7 ± 1.5	8.6 ± 2.4	< 0.001
MDA (µM)	33.2 (25.0–42.3)	34.0 (30.4–47.7)	0.771
PON1 (U/L)	24.4 (17.7–31.1)	42.2 (27.7–52.2)	< 0.001
Hypertension (%)	27.90	47.36	0.021

Comparisons between groups were performed using Independent Student’s t test or Mann–Whitney U test as appropriate. Continuous data are expressed as mean  ±  standard deviation (SD) or median and (interquartile range), categorical data as percentages

*BMI* body mass index; *WC* waist circumference; *WHR* waist to hip ratio; *TC* total cholesterol; *LDL-C* low density lipoprotein cholesterol; *HDL-C* high density lipoprotein cholesterol; *TG* triglycerides; *AST* aspartate amino transferase; *ALT* alanine amino transferase; *GGT* gamma glutamyl transferase; *LS* liver stiffness; *cIMT* carotid intima-media thickness; *MDA* malondialdehyde; *PON1* paraoxonase 1; *SOD* superoxide dismutase; *HOI* HDL oxidative index

P  <  0.05 was considered statistically significant

There was not any significant difference in the mean of HOI between NAFLD and control groups (P  =  0.405). We found that 61 (66.30%) controls and 30 (58.82%) patients had a preserved anti-oxidative function with an HOI  < 1, and 31 (33.69%) controls and 21 (41.17%) patients presented pro‐oxidative HDL serum measurements with an HOI  ≥  1 (P  =  0.468). We stratified the subjects into two groups, HOI  < 1 and HOI  ≥  1. The subjects with HOI  ≥ 1 had higher levels of MDA (P  =  0.042), lower PON1 activity (P  =  0.027), lower SOD activity (P  =  0.010), and higher cIMT values (P  =  0.022) (Additional file 1: Figure S1).

**Fig. 1:**
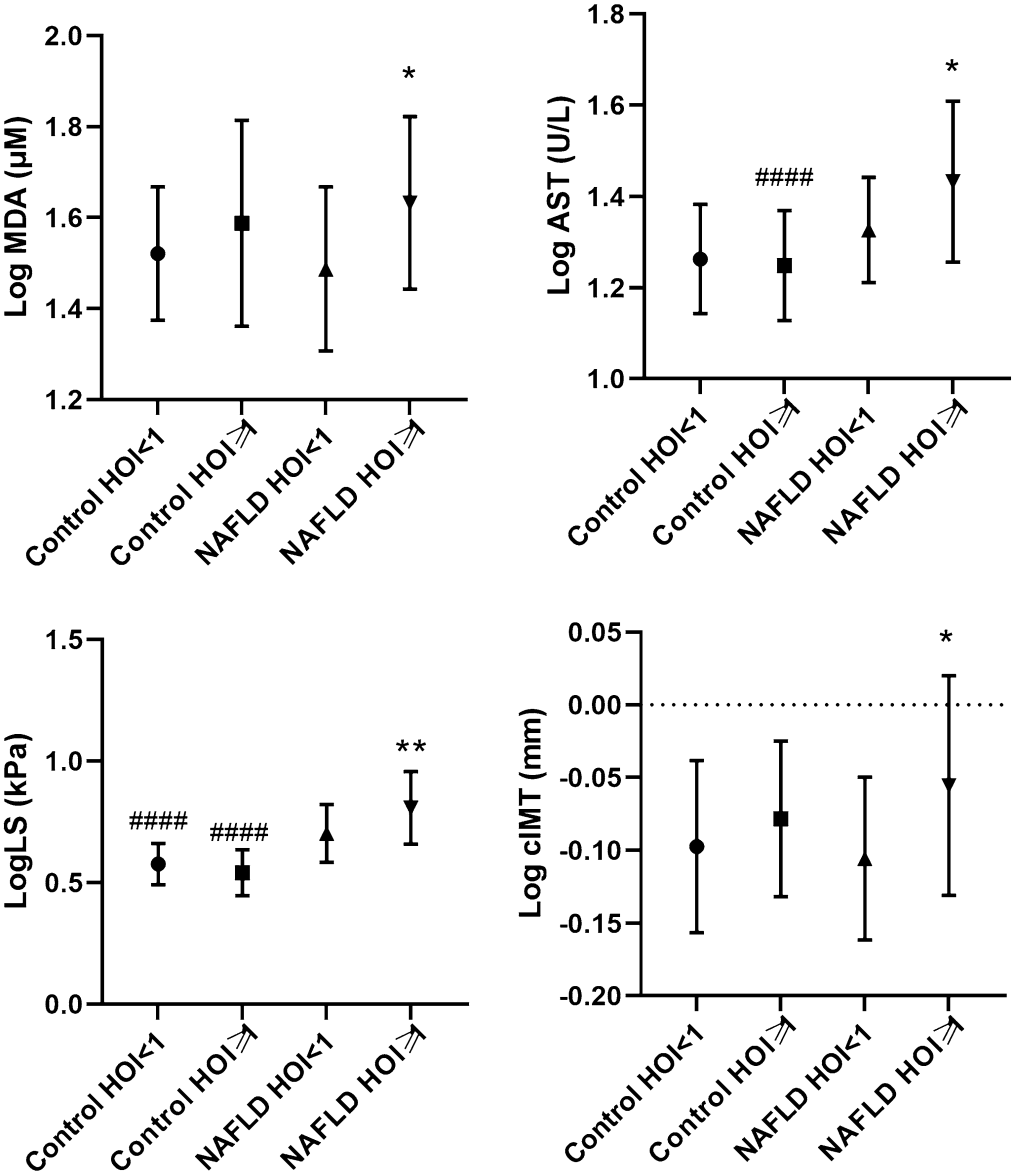
Comparison of AST, LS, cIMT, and MDA in the subjects stratified by a HOI below or above 1 and disease state. ANOVA with Tukey’s post-hoc test was used to compare among four groups. Data were presented as mean  ±  standard deviation (SD). *AST* aspartate amino transferase; *LS* liver stiffness, *cIMT* carotid intima-media thickness. P  < 0.05 was considered statistically significant. *Significant difference between HOI  <  1 and HOI  ≥ 1. #Significant difference between control and NAFLD groups. Significant differences were defined by P < 0.05 (*) or (#), P < 0.01 (**) or (##), and P < 0.0001 (****) or (####)

We further stratified the study population into 4 groups according to combined strata of status and HOI above and below 1 and compared MDA, antioxidant enzymes, NAFLD markers, and cIMT (Additional file 1: Table S1). The variables that were significantly different between HOI  < 1 and HOI  ≥ 1 subgroups of control or NAFLD groups were presented in Fig. 1. Interestingly, NAFLD patients with HOI  ≥ 1 had higher MDA (P  = 0.043), LS (P  = 0.008), AST (P  = 0.028) and cIMT (P  = 0.030) than the NAFLD patients with HOI  < 1.

Correlation analysis demonstrated that HDL antioxidant capacity (HOI) correlated positively with plasma MDA levels (r  = 0.298, P  = 0.001) and cIMT (r  = 0.197, P  = 0.025) in the whole population. There was a significant negative correlation between HOI and the SOD activity (r  =  − 0.242, P  =  0.004) in the whole population (Table 2).

**Table 2:** Correlation coefficients between HOI and metabolic and anthropometric parameters

Parameter	Correlation	P value
MDA	0.298	0.001
PON1	− 0.117	0.201
SOD	− 0.242	0.004
Age	0.056	0.503
cIMT	0.188	0.031
Visceral fat	0.01	0.909
WC	0.011	0.900
BMI	0.042	0.622
WHR	0.012	0.891
AST	0.059	0.488
ALT	0.067	0.432
GGT	0.075	0.374
LS	0.062	0.474
FBG	0.077	0.358
HDL-C	− 0.044	0.603
LDL-C	0.032	0.705
TG	− 0.009	0.919

*MDA* malondialdehyde; *PON1* paraoxonase 1; *SOD* superoxide dismutase; *cIMT* carotid intima-media thickness; *WC* waist circumference; *BMI* body mass index; *WHR* waist to hip ratio; *AST* aspartate amino transferase; *ALT* alanine amino transferase; *GGT* gamma glutamyl transferase; *LS* liver stiffness; *HDL-C* high density lipoprotein cholesterol; *LDL-C* low density lipoprotein cholesterol; *TG* triglycerides

## Discussion

Cardiovascular diseases are the main cause of death in NAFLD patients [27]. The impaired anti-oxidative capacity of HDL turned out to be a good predictor of cardiovascular disorders [5]. Our findings showed that impaired anti-oxidative capacity of plasma HDL was associated with higher levels of MDA, hepatic fibrosis markers, and cIMT in NAFLD.

A previous study on the anti-oxidative properties of HDL in patients with coronary syndrome found higher HOI in patients with acute coronary syndrome or stable coronary artery disease compared with controls [11]. Another study reported total HDL antioxidant capacity in systemic lupus erythematosus patients who have an increased risk of cardiovascular diseases were significantly reduced compared to the controls [28]. But there was no significant difference in anti-oxidative properties of HDL in MI patients with or without ST elevation and non-MI participants [10]. We also did not find a significant difference in HOI values between the NAFLD and the control groups. The frequency of HOI  ≥  1 was higher in NAFLD compared to the controls, albeit not significant. Consistently, it has been reported that impaired anti-oxidative capacity of plasma HDL was more frequent in women with polycystic ovary syndrome (PCOS) compared with the control group [13]. These conflicting results might be due to the different study populations and methods used in the studies.

In the current study, we found a higher level of MDA in the NAFLD group compared to the control group. In parallel, previous studies reported increased levels of MDA in the NAFLD patients compared to controls [23, 24, 29]. Additionally, a study showed increased levels of MDA in HDL subfractions isolated from the plasma of acute coronary syndrome patients compared to control subjects and it was along with a pro-inflammatory effect of HDL [30]. Consistently, we found higher levels of plasma MDA in the NAFLD patients with pro-oxidative HDL (HOI  ≥ 1) compared to the patients with HOI  < 1. Our findings also showed a significant positive correlation between HOI values and MDA levels. Moreover, our results showed higher levels of PON1 paraoxonase activities in the NAFLD patients than in the controls. Conversely, other studies showed lower PON1 serum concentration in the NAFLD group compared to the control group [31, 32]. However, in consistent with our findings, a large population study that used fatty liver index for the diagnosis of NAFLD showed that in men PON-1 activity was significantly higher in the NAFLD population compared to the non-NAFLD population [33]. PON1 has been shown to be critical for the anti-oxidative function of HDL particles. A previous study demonstrated a lower PON-1 activity in patients with an HOI  ≥ 1 than the subjects with an HOI  < 1 [4]. Although we found lower levels of PON1 paraoxonase activity in all subjects with HO  ≥ 1 compared to the subjects with HOI  < 1, we didn’t see any significant difference in PON1 between NAFLD patients with HOI  ≥ 1 and the patients with HOI  < 1. Considering SOD activity, we observed decreased activity of SOD in NAFLD patients. Similarly, other studies reported lower activity of SOD in NAFLD patients [23, 34, 35]. Our findings showed a lower SOD activity in all subjects with HOI  ≥ 1 compared to the subjects with HOI  < 1 but SOD was not significantly different in the NAFLD patients with HOI  ≥ 1 compared to the patients with HOI  < 1.

Moreover, we found that NAFLD patients with the impaired antioxidant function of HDL (HOI  ≥ 1) had higher AST and LS. Previous studies in NAFLD patients demonstrated the association of circulating oxidative stress biomarkers with disease severity [17]. In the light of the important role of oxidative stress in the progression of NAFLD, one can speculate that NAFLD patients with higher liver enzymes and liver fibrosis have higher levels of pro-oxidative and other detrimental factors which cause HDL dysfunctional. However, owing to the multiple anti-oxidative and anti‐inflammatory properties of HDL, the possibility that an impaired anti-oxidative capacity of HDL may affect disease severity in NAFLD patients cannot be ruled out.

Finally, we observed higher levels of cIMT in the subjects with HDL  ≥ 1 compared to the subjects with HOI  < 1. We also found a significant positive correlation between HOI and cIMT. However, a previous study on young adults did not find an association between the impaired anti-oxidative capacity of HDL and cIMT [26]. cIMT is considered a surrogate marker of cardiovascular risk [36]. Our findings regarding the association of HOI with cIMT support the notion that impaired anti-oxidative capacity of HDL can be a good predictor of cardiovascular issues. Of note, we found that NAFLD patients with the impaired antioxidant function of HDL (HOI  ≥ 1) had higher cIMT. This would suggest that the antioxidant function of HDL might be one of the potential mediators of the relationship between NAFLD and CVD.

There are some limitations to this study. The study was cross-sectional in nature, which does not provide information about possible causal relationships among NAFLD markers, HDL anti-oxidative capacity, and oxidative stress markers. So, further prospective studies are required to specifically address the potential role of HDL anti-oxidative function in the development and progression of NAFLD. We used apolipoprotein B–depleted plasma samples instead of isolated HDL to measure the anti-oxidative capacity of HDL. Polyethylene glycol precipitation is considered as a reproducible and rapid method to extract HDL from plasma samples [5, 11], but there is a possibility that anti-oxidative effects are influenced by other proteins rather than HDL associated proteins. However, a previous study showed comparable HOI values determined with apo B depleted sample and ultracentrifugation isolated HDL.

## Conclusions

In summary, our results showed that an impaired anti-oxidative capacity of HDL in NAFLD patients was associated with higher NAFLD markers. This association suggests either this disease state might attenuate the anti-oxidative effect of HDL or HDL anti-oxidative functionality might contribute to NAFLD pathogenesis.

## Supplementary Information

**Additional file 1: Figure S1.** Comparison of cIMT, plasma levels of MDA, PON1 and SOD in the subjects with preserved anti-oxidative HDL capacity (HOI < 1) and impaired anti-oxidative HDL capacity (HOI ≥ 1). The differences between two groups were analyzed by independent Student’s t test and were presented as mean ± SD or median (IQR). *MDA* malondialdehyde; *PON1* paraoxonase 1; *SOD* superoxide dismutase; *cIMT* carotid intima-media thickness. **Table S1.** Comparison of MDA, antioxidant enzymes, NAFLD markers and cIMT in the subjects stratified by a HOI below or above 1 and disease state. ANOVA was used to compare among four groups. Data were presented as mean ± standard deviation (SD). *AST* aspartate amino transferase; *LS* liver stiffness; *cIMT* carotid intima-media thickness; *MDA* malondialdehyde.

## Data Availability

The datasets used and/or analyzed during the current study are available from the corresponding author on reasonable request.

## Abbreviations

NAFLD: Non-alcoholic fatty liver disease
CVD: Cardiovascular disease
HDL: High-density lipoprotein
DCFDA: Dichlorofluorescein diacetate
PON1: Paraoxonase 1
HOI: HDL oxidative index
SOD: Superoxide dismutase (SOD)
MDA: Malondialdehyde (MDA)
CIMT: Carotid intima-media thickness
LDL-C: Low-density lipoprotein cholesterol
VAT: Visceral adipose tissue thickness
LS: Liver stiffness
FBG: Fasting blood glucose
TC: Total cholesterol
TG: Triglyceride
HDL-C: High-density lipoprotein cholesterol
ALT: Alanine amino transferase
AST: Aspartate amino transferase
GGT: Gamma glutamyl transferase
