# Supplementary material for: Association of anti-oxidative capacity of HDL with subclinical atherosclerosis in subjects with and without non-alcoholic fatty liver disease
Source: Diabetol Metab Syndr. 2021 Oct 26;13:121. doi: 10.1186/s13098-021-00741-5 (PMC8549143; doi:10.1186/s13098-021-00741-5)
Supplement: Supplementary file 1 — Additional file 1: Figure S1. Comparison of cIMT, plasma levels of MDA, PON1 and SOD in the subjects with preserved anti-oxidative HDL capacity (HOI < 1) and impaired anti-oxidative HDL capacity (HOI ≥ 1). The differences between two groups were analyzed by independent Student’s t test and were presented as mean ± SD or median (IQR). MDA malondialdehyde; PON1 paraoxonase 1; SOD superoxide dismutase; cIMT carotid intima-media thickness. Table S1. Comparison of MDA, antioxidant enzymes, NAFLD markers and cIMT in the subjects stratified by a HOI below or above 1 and disease state. ANOVA was used to compare among four groups. Data were presented as mean ± standard deviation (SD). AST aspartate amino transferase; LS liver stiffness; cIMT carotid intima-media thickness; MDA malondialdehyde. [file 13098_2021_741_MOESM1_ESM.docx]

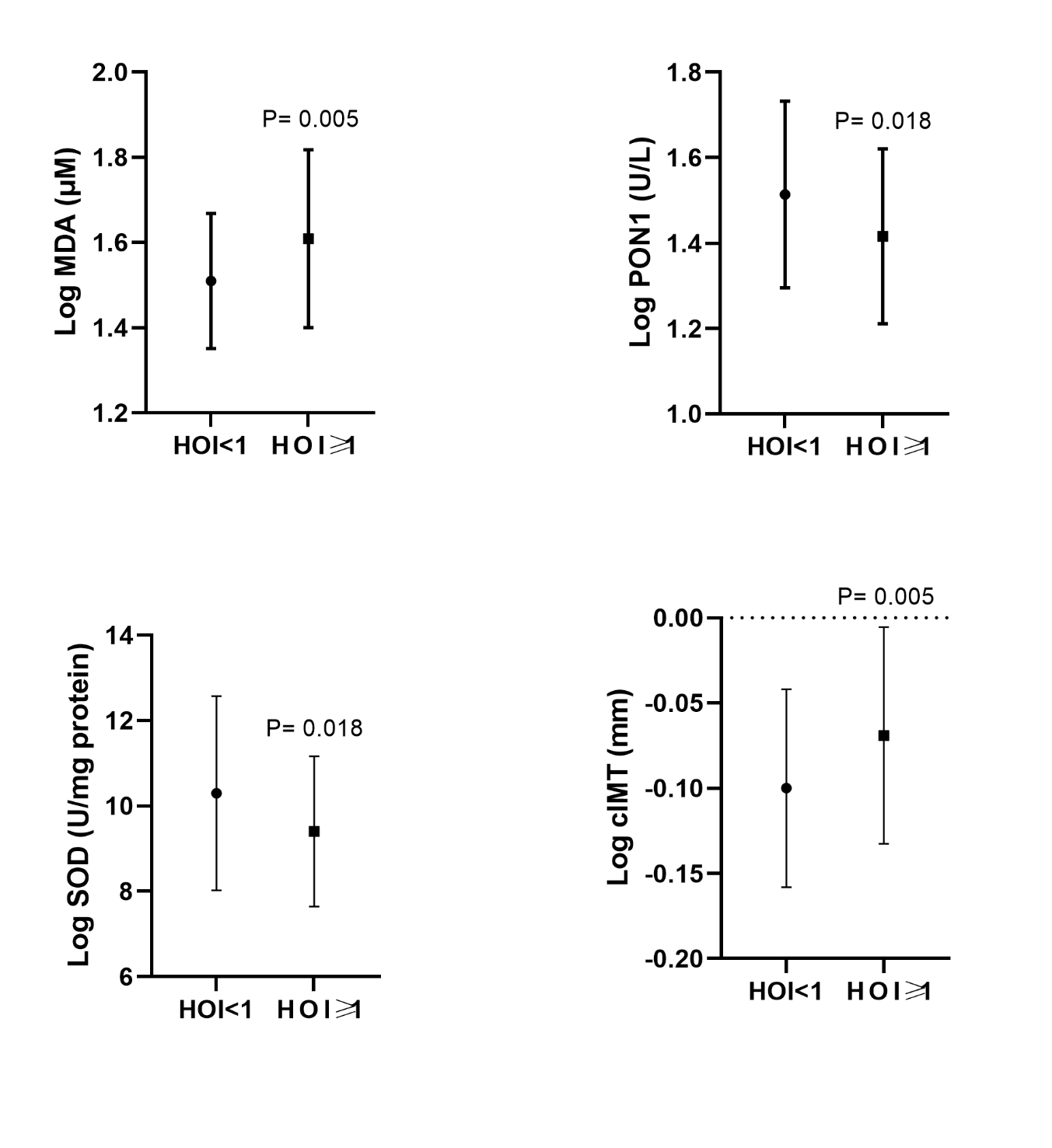


**Supplementary Figure 1**: Comparison of cIMT, plasma levels of MDA, PON1 and SOD in the subjects with preserved anti-oxidative HDL capacity (HOI<1) and impaired anti-oxidative HDL capacity (HOI≥1). The differences between two groups were analyzed by independent Student’s t-test and were presented as mean ± SD or median (IQR). MDA: malondialdehyde, PON1: Paraoxonase 1, SOD: superoxide dismutase, cIMT: Carotid intima-media thickness.

|  | Control with HOI<1 | Control with HOI ≥1 | NAFLD with HOI<1 | NAFLD with HOI ≥1 | P-value |
| --- | --- | --- | --- | --- | --- |
| Log MDA | 1.52 ± 0.14 | 1.58 ± 0.22 | 1.48 ± 0.18 | 1.63 ± 0.18 | 0.031 |
| SOD | 10.85 ± 1.65 | 10.42 ± 1.13 | 9.09 ± 2.90 | 7.91 ± 1.41 | <0.0001 |
| Log PON1 | 1.44 ± 0.20 | 1.35 ± 0.20 | 1.63 ± 0.19 | 1.51 ± 0.15 | <0.0001 |
| Log cIMT | -0.09 ± 0.05 | -0.07 ± 0.05 | -0.10 ± 0.05 | -0.05 ± 0.07 | 0.021 |
| Log AST | 1.26 ± 0.11 | 1.24 ± 0.12 | 1.32 ± 0.11 | 1.43 ± 0.17 | <0.0001 |
| Log ALT | 1.21 ± 0.21 | 1.15 ± 0.20 | 1.41 ± 0.16 | 1.55 ± 0.21 | <0.0001 |
| Log LS | 0.57 ± 0.08 | 0.54 ± 0.09 | 0.70 ± 0.11 | 0.80 ± 0.14 | <0.0001 |

Supplementary Table 1: Comparison of MDA, antioxidant enzymes, NAFLD markers and cIMT in the subjects stratified by a HOI below or above 1 and disease state. ANOVA was used to compare among four groups. Data were presented as mean ± standard deviation (SD). AST; aspartate amino transferase; LS: liver stiffness; cIMT: Carotid intima-media thickness; MDA: Malondialdehyde.
